# Ferulic acid attenuates high-fat diet-induced hypercholesterolemia by activating classic bile acid synthesis pathway

**DOI:** 10.3389/fnut.2022.976638

**Published:** 2022-09-21

**Authors:** Zhixin Luo, Mengqian Li, Jiachuan Yang, Jia Li, Yao Zhang, Fang Liu, Emad El-Omar, Lin Han, Ji Bian, Lan Gong, Min Wang

**Affiliations:** ^1^College of Food Science and Engineering, Northwest A&F University, Yangling, China; ^2^Microbiome Research Centre, St George and Sutherland Clinical School, University of New South Wales, Sydney, NSW, Australia; ^3^Kolling Institute, Sydney Medical School, Faculty of Medicine and Health, University of Sydney, Royal North Shore Hospital, St Leonards, NSW, Australia

**Keywords:** bile acid, cholesterol, CYP7A1, ferulic acid, hypercholesterolemia

## Abstract

Ferulic acid (FA), a natural phenolic phytochemical abundantly present in whole grains, displays promising therapeutic effects on hypercholesterolemia while its underlying mechanism not fully elucidated. This study aimed to investigate the cholesterol-lowering effect of FA in high-fat diet (HFD)-fed mice and its potential molecular mechanism. FA supplementation alleviated HFD-induced hypercholesterolemia (–13.2%, *p* < 0.05), along with increased excretion of bile acids (BAs) in feces (37.0%, *p* < 0.05). Mechanism studies showed that FA activated the expression of cholesterol 7α hydroxylase (CYP7A1), a rate-limiting enzyme in BA biosynthesis in the liver, which increased the BAs biosynthesis from cholesterol. Surprisingly, increased excretion of BAs in feces is a consequence, not a cause, of CYP7A1 activation. Furthermore, enterohepatic farnesoid X receptor (FXR) signaling is not involved in the activation of hepatic CYP7A1 by FA. In conclusion, FA activates CYP7A1 through non-FXR signaling, which on the one hand effectively prevents hypercholesterolemia, and on the other hand leads to secondary BAs elevation in plasma. The latter may be the key to the anti-obesity and hypoglycemic effects of FA.

## Introduction

Ferulic acid (FA, 4-hydroxy-3-metoxybenzene acrylic acid), the most representative phenolic acid in whole grains, is widely present in rice (1), rye (2), wheat (3) and barley (4). It has also been reported that FA exhibits cardiovascular protection (5) with anti-obesity beneficial potential (6).

Hypercholesterolemia is the hallmark of abnormal cholesterol metabolism (7), which is a major risk factor for atherosclerosis (8–10). Although the anti-hypercholesterolemia beneficial potential of FA has been reported in humans (11), *ApoE*-/- mice (12) and rats (13), the underlying mechanisms have not been sufficiently elucidated.

Bile acids (BAs) are the end products of cholesterol catabolism in the liver (14). The synthesis of BAs is the most important mechanism for degrading and eliminating cholesterol (15). In the classical BA biosynthesis pathway, cholesterol 7α hydroxylase (CYP7A1) is the rate-limiting enzyme that converts cholesterol to 7α-hydroxycholesterol at the initiatory step (16). The mice overexpressing CYP7A1 have an increased level of total BAs in fecal (17). Besides, in enterohepatic circulation of BAs, about 95% of the BAs are reabsorbed in the ileum and released into the portal vein and redirected to the liver for recycling. If BAs reabsorption is inhibited (e.g., with BAs sequestrants), level of total BAs in fecal will also increase. Some studies have reported that FA supplementation increases the excretion of BAs in feces (12, 13), however, how FA affects fecal BAs excretion has not been reported. Therefore, we have conducted in-depth research on this issue.

In this study, we focused on the mechanism by which the FA reduces cholesterol levels. It was found that FA attenuates HFD-induced hypercholesterolemia by activating CYP7A1, the rate-limiting enzyme of classic BAs biosynthesis, via a non-FXR signaling. And the increased excretion of BAs in feces is the result of activation of CYP7A1. In addition, activation of CYP7A1 in the liver leads to the accumulation of secondary BAs in plasma, which may contribute to the hypoglycemic and anti-obesity activities of FA. This study will help to further reveal the potential mechanism of regulation of FA on glucose and lipid metabolism through BAs biosynthesis.

## Materials and methods

### Chemicals and reagents

FA (purity ≥ 99%) was purchased from Solarbio Science and Technology Co., Ltd. (Beijing, China). Primers (Table 1) used for qRT-PCR were ordered from Sangon Biotechnology Ltd. (Shanghai, China). Commercial detection kits for alanine transaminase (ALT), aspartate transaminase (AST), total cholesterol (TC), triglycerides (TG) were obtained from Changchun Huili Biotech Co., Ltd. (Jilin, China). Mouse Insulin Elisa Kit (ED-20778) was obtained from Lunchangshuo Biotech Co., Ltd. Alkaline phosphatase (ALP) kit, γ-glutamytransferase (GGT) kit and total BA kit (E003-2-1) were obtained from Nanjing Jiancheng Bioengineering Institute. Other chemicals and reagents were purchased from Sigma-Aldrich (Germany) unless specifically stated.

**TABLE 1:** Primer sequences used for qRT-PCR.

Gene (transcript)	Sequences (5′–3′)	Products length/bp	Tm/°C
*Actb* (NM_007393.5)	GGCTCCTAGCACCATGAAG	190	58
	CGCAGCTCAGTAACAGTCC		
*Cyp7a1* (NM_007824.3)	TGCTTCCTGCTTTGCCTTG	224	58
	ACCAGGAGACCCATCATTC		
*Cyp7b1* (NM_007825.4)	AGCCCTCTTTCCTCCACTC	127	57
	CAGCTTCTTCCCTCCTTTG		
*Cyp27a1* (NM_024264.5)	CGATGGCTGAGGAAGAAAG	165	56
	AGCCAGGGCAATCTCATAC		
*Cyp8b1* (NM_010012.3)	CCATAAGACGCCATCCCTC	149	57
	GCAAGATGCCATCTGACTC		
*Cyp2c70* (NM_145499)	CGTAAGACAACGCAGGATG	130	56
	GGCCAGGATCAAACTTCTC		
*Slc51a* (NM_145932.3)	CTCCATCTTGGCTAACAG	163	52
	CTCATACCCAACCTTGTC		
*Slc51b* (NM_178933.2)	AGAAGATGCGGCTCCTTG	192	57
	AGGAAGACCTGGCTGTTG		
*Slc10a2* (NM_011388.3)	GATCTCAACCTGGTGTTC	165	53
	AAATGATGGCCTGGAGTC		
*Nr1h4* (XM_030244963.2)	AGCGATCGTCATCCTCTC	133	56
	GAAATGCTGCGGGTTCTC		
*Nr0b2* (NM_011850.3)	TGGGTCCCAAGGAGTATGC	138	59
	ACCAGGGCTCCAAGACTTC		
*Ffg15* (NM_008003.2)	GGACGGCAAGATATACGG	110	55
	GGAGATGGTGCTTCATGG		

### Animal experiment

Four-week-old male C57BL/6J mice were obtained from the Experimental Animal Center of Xi’an Jiaotong University (Xi’an, China). All mice were housed in the Northwest A&F University animal facility under a constant temperature (22 ± 2°C) and with 12-h light/dark cycles. The mice were fed a normal chow diet for an acclimatization period of 1 week after their arrival. At 5 weeks of age, they were randomly divided into 3 groups of 10 mice per group and fed with control diet (CON group), high-fat diet (HFD group), or HFD plus FA (100 mg/kg of body weight) (HFD + FA group) for 12 weeks continuously. The control diet (TP23402, TROPHIC Animal Feed High-tech Co., Ltd., Nantong, China) contains 10.0% kcal from fat, 14.1% kcal from protein, and 75.9% kcal from carbohydrate, while the HFD (TP23400, TROPHIC Animal Feed High-tech Co., Ltd., Nantong, China) contains 60.0% kcal from fat, 14.1% kcal from protein, and 25.9% kcal from carbohydrate. FA dissolved in sodium carboxymethylcellulose (CMC-Na, 0.3% w/v) was orally administered to the HFD + FA mice via a gastric tube, while the CON group and the HFD group were also received equal volume of vehicle CMC-Na. Body weight and food intake were recorded daily. At designated experimental endpoints, all mice were anesthetized with isoflurane and cervical dislocation after a 12-h fast. Serum and tissue samples were then collected by either snap-frozen in liquid nitrogen and store at -80°C or directly stored in 4% paraformaldehyde for histological analysis. All animal experimental procedures were followed using the Guide for the Care and Use of Laboratory Animals: Eighth Edition (ISBN-10: 0-309-15396-4). We have complied with all relevant ethical regulations for animal experiment. All animal studies were approved by the Institutional Animal Care and Use Committee of the Northwest A&F University (Permission ID: 20200528-010), and therefore the animal experiments were carried out in accordance with all animal care regulations, guidelines, and standards.

### Dosage information

The dosage of FA used in this study was calculated based on pilot experiments and the reference, at which no toxicity was observed in human (18). The dose of 100 mg/kg of body weight per day for mice was equivalent to 500 mg FA per day for a mean human weight of 60 kg according to the formula for dose translation based on body surface area: mouse equivalent doses (mg kg^–1^) = human dose (mg kg^–1^) × (Km- adult/Km- mouse), Km factor of 3 and 37 for adult mouse (0.02 kg) and adult human (60 kg), respectively (19). The use of a single dose in this trial helped control experimental costs.

### Metabolic cage studies

Mice were individually housed in metabolic cage at room temperature (22 ± 2°C) and allowed to acclimate for 24 h. After acclimatization, the mice were gavaged with FA (100 mg/kg) every morning for 3 days. Oxygen consumption (VO_2_), carbon dioxide production (VCO_2_), heat value and respiratory exchange ratio (RER) were measured every 5 min. The RER was derived from the ratio of VCO_2_ to VO_2_, and energy expenditure was determined as (3.815 + 1.232 × RER) × VO_2_ and expressed as kcal h^–1^. Total body weight was used as the covariate in the analysis.

### Biochemical analysis of serum and tissue samples

The serum concentrations of TC, TG, ALT, AST, ALP, GGT and insulin were determined using an automatic biochemical analyzer (Chemray 240, Rayto Life and Analytical Sciences Co., Ltd., Shenzhen, China) according to the manufacturer’s instructions. For liver tissues, TC level were normalized to those of the protein concentrations in the initial homogenate, as determined by protein quantification using a BCA Protein Assay Kit (Beyotime Technology, Shanghai, China). Fecal total BAs were quantified using Total BA kits following manufacturer protocol.

### Liver immunostaining

The murine liver tissue sections were prepared as described in Luo et al. (20). For immunohistochemistry (IHC) staining, the samples were incubated with antibodies against CYP7A1 (1:50 dilution; Solarbio Technology Co., Ltd., Beijing, China). Sections were then washed in PBS three times and incubated with secondary antibodies goat anti-rabbit IgG-HRP (1:500 dilution; Wuhan Servicebio Technology Co., Ltd., Wuhan, China). The sections then were stained with DAB according to the manufacturer’s protocol. After mounting, the sections were visualized and photographed using an optical microscope with camera (Olympus, Tokyo, Japan) at a 100 × magnification.

### LC-MS/MS-based metabolomics analysis

The murine liver tissues were weighed and freeze dried. The lyophilized samples were ground in a 2 mL Eppendorf tube containing 5 mm tungsten beads for 1 min at 65 Hz in a Grinding Mill (Wuhan Servicebio Technology Co., Ltd., Wuhan, China). Metabolites were extracted using 1 mL precooled mixtures of methanol, acetonitrile and water (v/v/v, 2:2:1) and then placed for 1 h ultrasonic shaking in ice baths. Subsequently, the mixture was centrifuged at 14,000 g for 20 min at 4°C. The supernatants were recovered and concentrated to dryness in vacuum. Metabolomics profiling was analyzed using an ultra-high-performance liquid chromatography coupled with electrospray ionization quadrupole time-of-flight mass spectrometry (UHPLC-ESI-Q-TOF-MS) system: UHPLC, 1290 Infinity LC (Agilent Technologies, Santa Clara, CA, USA) coupled with TripleTOF 5600 (AB Sciex, Framingham, MA, USA). The hydrophilic interaction liquid chromatography (HILIC) separation, MS data acquisition and identification of metabolites were performed as described in Han et al. (21). SIMCAP software (Version 14.1, Umetrics, Umeå, Sweden) was used for all multivariate data analyses and modeling. Data were mean centered using Pareto scaling. Models were built on principal component analysis (PCA), orthogonal partial least-square discriminant analysis (PLS-DA) and partial least-square discriminant analysis (OPLS-DA). All the evaluated models were tested for over fitting with methods of permutation tests.

### Kyoto encyclopedia of genes and genomes enrichment analysis

To identify the perturbed biological pathways, the differential metabolite data were extracted from metabolomics analysis to perform kyoto encyclopedia of genes and genomes (KEGG) pathway analysis using KEGG database.^1^ KEGG enrichment analyses were carried out with the Fisher’s exact test. FDR correction for multiple testing was also performed.

### Quantitative real-time PCR

Total RNA was extracted from the murine liver tissue using AG RNAex Pro Reagent (Accurate Biotechnology Co., Ltd., Hunan, China) following the manufacturer’s instructions. For quantitative real-time PCR (qRT-PCR) analysis, the first-strand cDNA was obtained using Evo M-MLV RT Kit with gDNA Clean for qPCR (Accurate Biotechnology Co., Ltd., Hunan, China), and then were subjected to quantification of the mRNAs with β-actin as an endogenous control on the Bio-Rad CFX96 Touch™ Real Time PCR Detection System (Bio-Rad, United States). The qRT-PCR reaction consisted of 10 μL 2 × SYBR^®^ Premix Ex Taq™ II (CWBIO Bio., China), 0.8 μL specific forward/reverse primer (10 μM), 1 μL cDNA, and ddH_2_O to a final volume of 20 μL. The quantitative PCR was performed using the following conditions: 95°C for 5 min, 40 cycles of 95°C for 5 s, and the optimized annealing temperature for 30 s. Each group had at least 6 samples, and all reactions were performed in triplicate for each sample.

### Quantitative analysis of bile acids

BAs were extracted from murine serum samples with 200 μL methanol and analyzed on an LC-ESI-MS/MS system (UHPLC, ExionLC AD; MS, Applied Biosystems 6500 Triple Quadrupole) as described in Garcia-Canaveras et al. (22).

### Intestinal microbiome analysis by 16S rRNA amplicon sequencing

For intestinal microbiome analysis, 16S rRNA amplicon sequencing was performed. Total genomic DNA were extracted from murine cecal contents using the E.Z.N.A. Stool DNA Kit (D4015, Omega, Inc., United States) according to the manufacturer’s instructions. The V3-V4 region of the 16S rRNA gene was amplified using the forward primer 5′-CCTACGGGNGGCWGCAG-3′ and reverse primer 5′- GACTACHVGGGTATCTAATCC -3′. The amplicons were purified using Agencourt AMPure Beads (Beckman Coulter, Indianapolis, IN, USA) and quantified using the PicoGreen dsDNA Assay Kit (Invitrogen, Carlsbad, CA, USA). After the individual quantification step, the amplicons were sequenced using an Illumina NovaSeq platform with MiSeq Reagent Kit V3 at LC-Biotechnology Co., Ltd. (Hangzhou, China). The Quantitative Insights into Microbial Ecology (QIIME2, v1.8.0) pipeline was employed to process the sequencing data and the chimera-free sequences were aligned with the SILVA database at 97% identity. Other diagrams were implemented using the R package (v3.5.2).

### Western blot analysis

Preparation of mouse liver tissue lysate and western blot analysis were as previously described (20). The difference is that the PVDF membranes were incubated overnight at 4°C with the antibody against CYP7A1 followed by the secondary antibody (Solarbio Technology Co., Ltd., Beijing, China).

### Statistical analysis

All numerical values were presented as mean ± SEM. Statistical analyses were conducted using an unpaired *t*-test and one-way analysis of variance (ANOVA), followed by Tukey’s *post hoc* comparison test. The discriminating metabolites were obtained using a statistically significant threshold of variable influence on projection (VIP) values obtained from the OPLS-DA model and two-tailed Student’s *t*-test (*p*-value) on the normalized raw data at univariate analysis level. Metabolite changes with VIP value greater than 1.0 and *p*-value less than 0.05 were considered as statistically significant. Fold change was calculated as the logarithm of the average mass response (area) ratio between two arbitrary classes. On the other side, the identified differential metabolites were used to perform cluster analyses with R package. Figures were generated by Prism v8.2.1 (GraphPad Software Inc., USA). *p < 0.05; ^**^p < 0.01; ^***^p < 0.001; ^****^p < 0.0001.

## Results

### Ferulic acid supplementation alleviates hypercholesterolemia and increases excretion of total bile acids in feces

**FIGURE 1:**
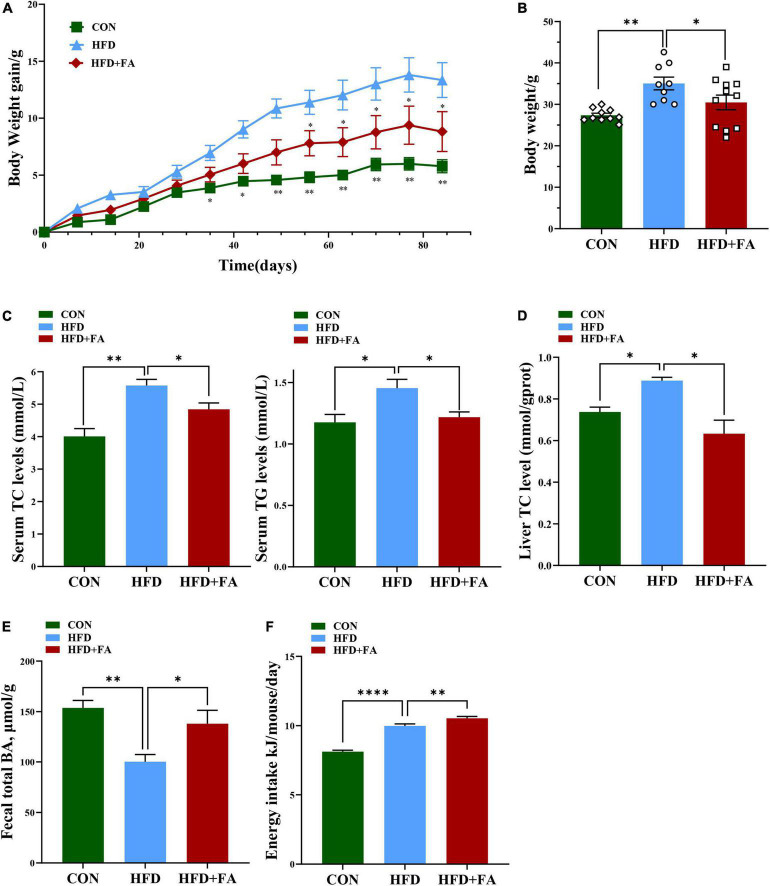
FA alleviates hypercholesterolemia and increases excretion of total bile acids in feces. **(A)** Body weight gain change curve (12 weeks). **(B)** Body weight (g). **(C)** Serum TC and TG levels (mmol/L). **(D)** Hepatic TC levels. **(E)** Fecal total BAs level. **(F)** Energy intake (Kj/mouse/day). Data are shown as mean ± SEM (*n* = 9–10). Statistical analysis was performed using one-way ANOVA followed by Dunnett’s multiple comparison test. *p < 0.05; ^**^p < 0.01; ^****^, *p* < 0.0001. CON, control diet group; HFD, high-fat diet group, HFD + FA, high-fat diet with 100 mg kg/bw/day FA group.

To investigate the effect of FA on hypercholesterolemia, male C57BL/6 mice fed with HFD were treated with 100 mg/kg bw/day of FA in the HFD + FA group by oral gavage daily for a period of 12 weeks. The CON group and the HFD group were also administered with equal volume of vehicle CMC-Na. In terms of the initial body weight at week 5, no significant difference was observed among these three groups (data not shown). As shown in Figure 1A, the body weight continued to increase throughout the experiment. Compared with the CON mice fed with control diet, the HFD mice showed higher body weight gain from 5 weeks onward (*p* < 0.05). After 9 weeks of FA treatment, the body weight gain of the HFD + FA mice was less than that of the HFD mice (*p* < 0.05). At the end of the 12-week FA-treatment, the body weight gain of the HFD + FA group was significantly reduced by 13.1% (*p* < 0.05) compared to the HFD group (Figure 1B). Notably, the HFD mice displayed dyslipidemia, which is characterized by an increment in serum concentrations of TC and TG in comparison with the CON mice (*p* < 0.05), which were significantly reduced in the HFD + FA mice by approximately 13.2 and 16.3%, respectively (Figure 1C). In addition, FA supplementation decreases the hepatic TC level and increases the excretion of total BAs in feces (Figures 1D, E). Collectively, these data demonstrate that FA supplementation at 100 mg/kg bw/day dose can significantly attenuate hypercholesterolemia and obesity in HFD-fed mice. Interestingly, the energy intake of the HFD + FA mice increased by approximately 0.542 kJ/day/mouse (*p* < 0.01) compared to the HFD group (Figure 1F), suggesting that the FA-mediated reduction of body weight gain and serum TC level was not due to energy intake decrease.

### Ferulic acid supplementation improves hepatic metabolism

**FIGURE 2:**
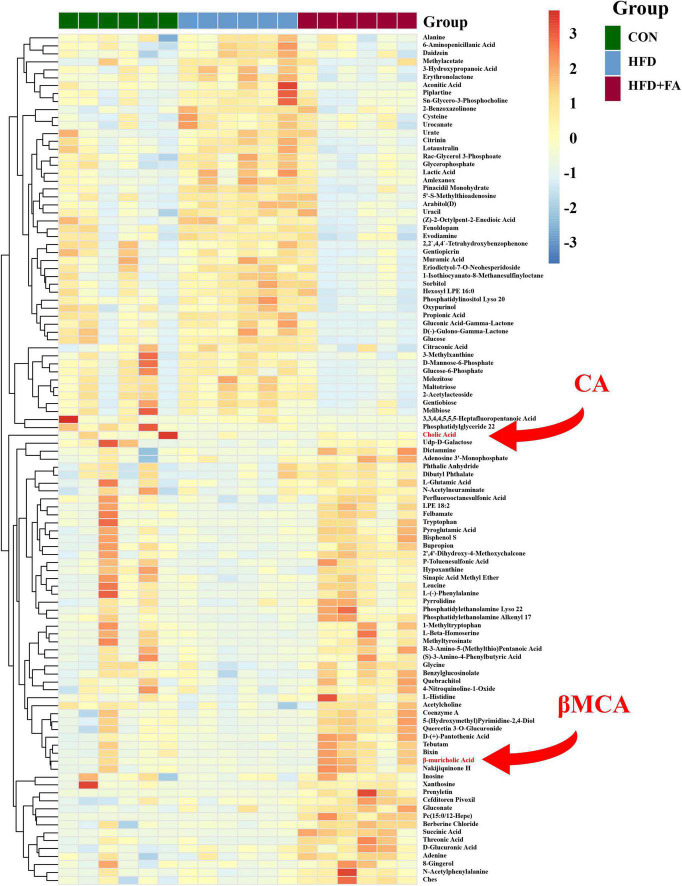
Effects of FA supplementation on hepatic metabolite profile in HFD-fed mice. The hepatic metabolomics analysis was performed in the CON, HFD and HFD + FA mice. Heatmap of representative differential metabolites from the CON, HFD and HFD + FA mice (*n* = 6). Colors represent the contents of differential metabolites with red indicating a high content and blue indicating a low content. The red arrow indicates metabolites CA and βMCA. CON, control diet group; HFD, high-fat diet group; HFD + FA, high-fat diet with 100 mg/kg bw/day FA group.

To further investigate the mechanism by which FA reduces hepatic cholesterol level, the liver metabolome was analyzed with an untargeted metabolomics approach using the UPLC-ESI-MS platform in both negative and positive modes. Three-dimensional data involving the peak number, sample name, and normalized peak area were analyzed with SIMCA14.1 software package (Umetrics, Umea, Sweden) for PCA and OPLS-DA. A total of 702 discriminant metabolite species were identified finally. OPLS-DA showed that the distributions of origin data and the FA-treated group were clearly discriminated from the HFD group, indicating that FA supplementation had a substantial effect on hepatic metabolite profile (Supplementary Figure 1A). Analysis of quality control (QC) samples confirmed low system variance with low coefficient of variation. To further verify significant variables, the first principal component of VIP was calculated, in which VIP value > 1 or *p* < 0.05 of Student’s *t*-test was considered as statistical difference. Metabolomics analysis successfully identified 72 differential metabolites between the HFD group and the CON group (*p* < 0.05), as well as 107 differential metabolites between the HFD group and the HFD + FA group (*p* < 0.05), Among which 57 metabolites were increased while 50 metabolites were decreased in the FA-treated mice in comparison to the HFD mice (Figure 2). Further analysis of online database KEGG pathway was utilized to explore the potential mechanism of FA intervention in liver metabolism. The results showed that FA can regulate pyrimidine metabolism, insulin signaling, glycerophospholipid metabolism, galactose metabolism and bile secretion (Supplementary Figure 1B).

### Ferulic acid supplementation increases primary bile acid biosynthesis in the liver

**FIGURE 3:**
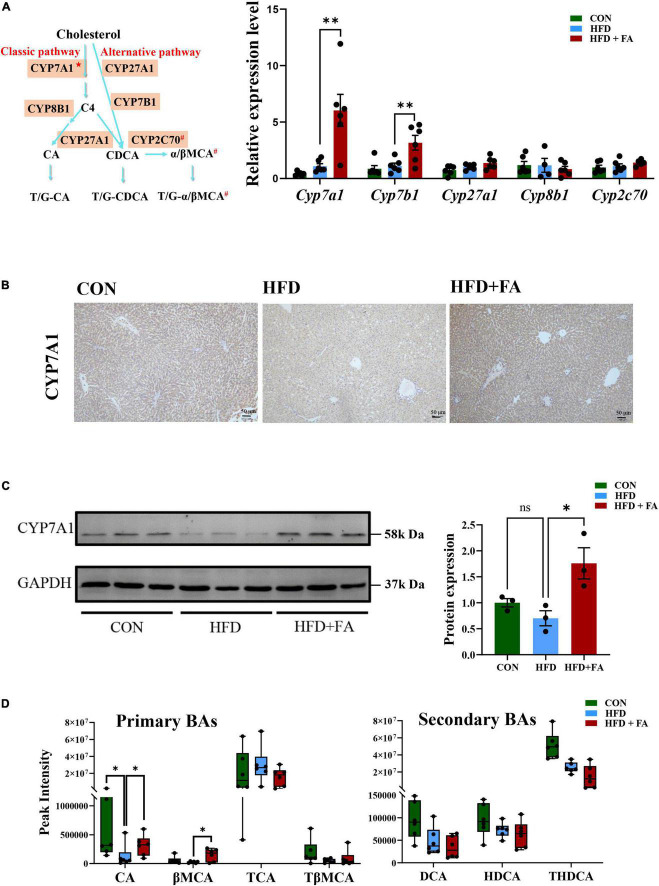
FA increases bile acid biosynthesis in the liver. **(A)** Schematic representation of synthetic pathways of primary BAs (left) and the mRNA levels of 5 key genes involved in the BAs biosynthesis including *Cyp7a1*, *Cyp7b1*, *Cyp27a1*, *Cyp8b1*, and *Cyp2c70* (right) in the livers of the CON, HFD and HFD + FA mice (*n* = 6). The relative mRNA levels were analyzed referring to the 2-ΔΔCT method and normalized to the Actb gene. C4: 7α-hydroxy-4-cholesten-3-one; CA: cholic acid; CDCA: chenodeoxycholic acid; α/βMCA: α/β- muricholic acid; ★The rate-limiting enzyme in BAs biosynthesis; ^#^Only in mice. **(B)** Representative images showing IHC staining of CYP7A1 protein in the livers of the CON, HFD and HFD + FA mice. Scale bar, 50 μm. **(C)** Western blot analysis of hepatic CYP7A1 protein in the CON, HFD, and HFD + FA mice (left, *n* = 3), and quantification analysis using ImageJ (right). **(D)** Relative levels of 7 primary and secondary BAs (CA, βMCA, TCA, TβMCA, DCA, HDCA, THDCA) identified in the livers of the CON, HFD, and HFD + FA mice (*n* = 6). Data are presented as mean ± SEM. *p < 0.05, ^**^p < 0.01, ns, no significance vs. HFD group. CON, control diet group; HFD, high-fat diet group; HFD + FA, high-fat diet with 100 mg/kg bw/day FA group.

The conversion of cholesterol to BAs in the liver is the predominant pathway for the elimination of cholesterol from the body. Therefore, we determined the expression of the key genes participated in BAs metabolism (*Cyp7a1, Cyp7b1, Cyp27a1, Cyp8b1, and Cyp2c70*, shown as Figure 3A left) using qRT-PCR. The results showed that FA supplementation in the HFD + FA group remarkably enhanced the mRNA levels of *Cyp7a1* (5.5-fold increase, *p* < 0.01) and *Cyp7b1* (2.8-fold increase, *p* < 0.01) as compared with the HFD group (Figure 3A right), while no significant changes in the mRNA levels of *Cyp27a1, Cyp8b1, and Cyp2c70* were observed. This upregulation of CYP7A1, the rate-limiting enzyme for BAs biosynthesis in the liver, was further confirmed by IHC (Figure 3B) and Western blot analysis (Figure 3C). Next, we measured the hepatic BAs profiles in the three groups of mice. Of the primary BAs detected in the liver, the concentrations of cholic acid (CA) and β-muricholic acid (βMCA) in the HFD + FA group were 2.1-fold and 6.0-fold higher than the HFD group, respectively (Figure 3D left). However, no significant changes were observed for the other primary and secondary BAs (TCA, TβMCA, DCA, HDCA, THDCA) detected (Figure 3D right). Taken together, the results indicate that FA promotes primary BAs biosynthesis.

### Ferulic acid supplementation increases bile acid reabsorption

**FIGURE 4:**
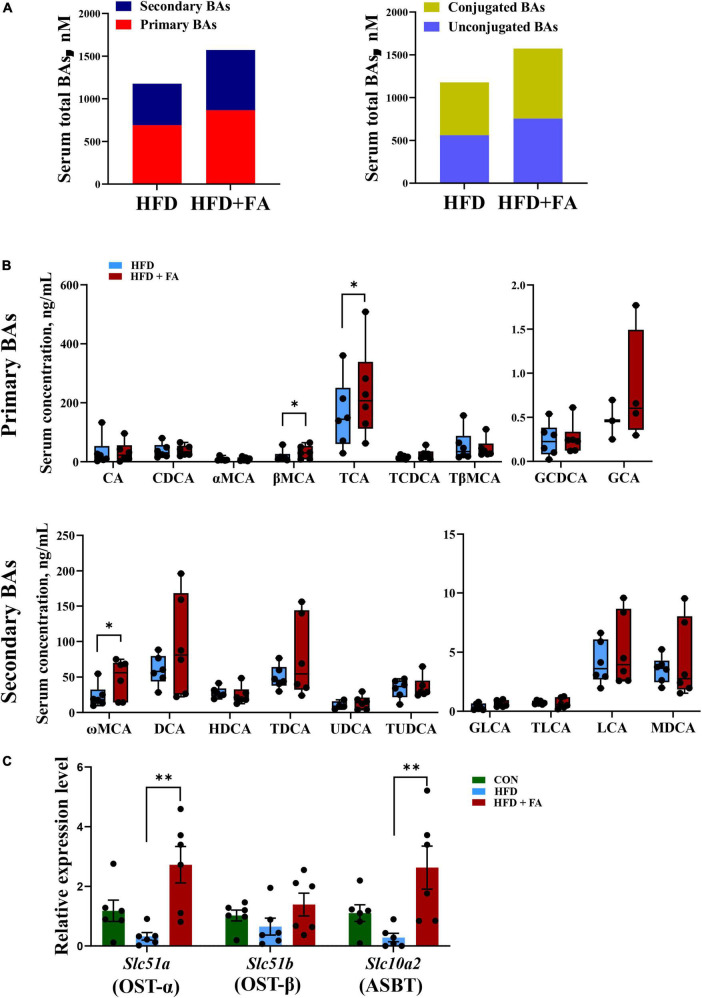
FA supplementation increases BAs reabsorption. **(A)** Serum total pools of primary BAs, secondary BAs, conjugated BAs and unconjugated BAs in the HFD and HFD + FA mice. **(B)** Serum BAs profiles of 9 primary BAs and 10 secondary BAs in the HFD and HFD + FA mice (*n* = 6). **(C)** The mRNA levels of OSTα, OSTβ and ASBT in the ileum samples from the CON, HFD, and HFD + FA mice (*n* = 6). Data are presented as mean ± SEM. *p < 0.05, ^**^p < 0.01. CON, control diet group; HFD, high-fat diet group; HFD + FA, high-fat diet with 100 mg/kg bw/day FA group.

To clarify the relationship between hepatic BA biosynthesis and fecal BA excretion, an UHPLC-ESI-MS/MS based targeted metabolomics approach was used to analyze the serum BAs profile. The results revealed that FA supplementation remarkably enhanced the size of serum BAs pools by 33.5%. Specifically, primary BAs and secondary BAs were increased by 25.3 and 45.2%, while conjugated BAs and unconjugated BAs were increased by 34.5 and 32.7% in the HFD + FA group as compared with the HFD group (Figure 4A). Detailed serum profile of 9 primary BAs and 10 secondary BAs showed that the levels of βMCA, tauro-cholic acid (TCA) and ω-muricholic acid (ωMCA) were dramatically elevated in the HFD + FA mice compared to the HFD mice (Figure 4B). Of note, there was no significant difference of serum AST, ALT, ALP and GGT activity between HFD group and HFD + FA group (Supplementary Figure 2), but the mRNA levels of key genes involved in BAs reabsorption in ileum including *Slc51a* and *Slc10a2* increased significantly in the HFD + FA group (Figure 4C). These results suggest that the increase of serum BAs pools is due to the increased BAs reabsorption rather than hepatotoxicity of FA.

### Enterohepatic farnesoid X receptor signaling is not involved in the activation of hepatic cholesterol 7α hydroxylase by ferulic acid

**FIGURE 5:**
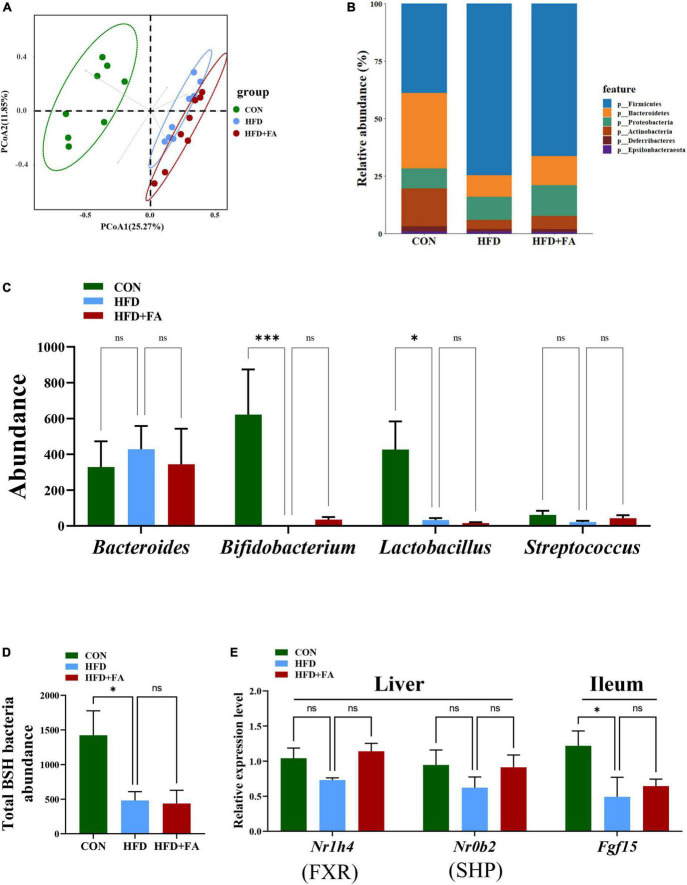
Enterohepatic FXR signaling is not involved in the activation of hepatic CYP7A1 by FA. **(A)** PCoA analysis of fecal microbiome based on unweighted UniFrac metrics at the operational taxonomic unit (OTU) level for all samples from the CON, HFD and HFD + FA mice (*n* = 8). **(B)** Relative abundances at phylum level. **(C)** The abundance of BSH-enriched bacterial genera including Bacteroides, Bifidobacterium, Lactobacillus, and Streptococcus in feces from the CON, HFD and HFD + FA mice (*n* = 8). **(D)** Abundance of total BSH enriched bacteria in feces from the CON, HFD, and HFD + FA mice (*n* = 8). **(E)** The mRNA level of FXR and SHP in the liver and Fgf15 in the ileum (*n* = 6). Data are presented as means ± SEM. *p < 0.05, ^***^p < 0.001, ns, no significance vs. HFD group. CON, control diet group; HFD, high-fat diet group; HFD + FA, high-fat diet with 100 mg/kg bw/day FA group.

Based on the important role of FXR signaling in BAs homeostasis and the possibility that gut microbiota may influence FXR signaling, we detect the abundance of BSH-enriched bacteria in feces by 16S rRNA amplicon sequencing. Unweighted UniFrac metrics-based principal coordinate analysis (PCoA) revealed a distinct clustering of community’s structure of fecal microbiota for each group (Figure 5A), although the FA-induced microbiota shift (HFD + FA vs. HFD) was smaller than the HFD-induced shift (HFD vs. CON). At the phylum level, no significant structural shifts of the gut microbiota were observed between the groups of HFD and HFD + FA, despite the difference between the groups of CON and HFD (Figure 5B). FA supplementation in the HFD + FA mice did not change the abundance of BSH-enriched bacterial genera including *Bacteroides*, *Bifidobacterium, Lactobacillus*, and *Streptococcus* comparing with the HFD mice (Figure 5C), whilst the total abundance of BSH-enriched bacterial genera in the HFD group markedly lower than that in the CON group (Figure 5D). Furthermore, FA supplementation did not cause significant changes in the mRNA levels of hepatic SHP and ileal FGF15 (Figure 5E) and the ratio of *Firmicutes*/*Bacteroidetes* (Supplementary Figure 3). In brief, these results suggest that the activation of BAs biosynthesis by FA is not through enterohepatic FXR signaling.

### Ferulic acid supplementation decreases fasting glucose and increases energy expenditure

**FIGURE 6:**
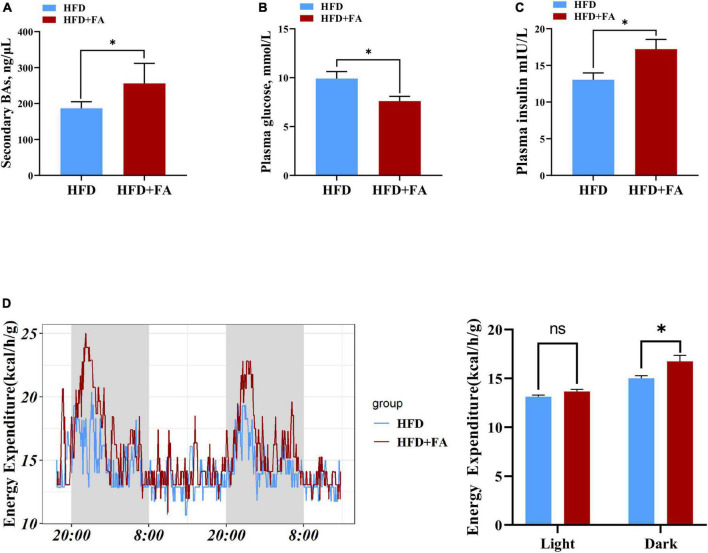
FA decreases fasting glucose and increases energy expenditure. **(A)** Serum total secondary BAs in the HFD and HFD + FA mice (*n* = 6). **(B)** Plasma insulin level after 6 h fasting in the HFD and HFD + FA mice (*n* = 8). **(C)** Plasma glucose level after 6 h fasting in the HFD and HFD + FA mice (*n* = 8). **(D)** Energy expenditure of mice from HFD and HFD + FA groups (*n* = 4). Data are presented as mean ± SEM. *p < 0.05, ns, no significance. HFD, high-fat diet group; HFD + FA, high-fat diet with 100 mg/kg bw/day FA group.

Given that the concentration of serum total secondary BAs in HFD + FA group was higher than that in HFD group (Figure 6A) and the important role of BAs in glucose homeostasis and energy metabolism, we next assessed the effect of FA on fasting glucose on energy expenditure. The results revealed that FA treatment resulted in a significantly increased serum insulin level (*p* < 0.05) in HFD-fed mice (Figure 6B), which was accompanied by a significant decrease in fasting glucose levels (9.911 ± 0.7142 mmol/L in HFD group, 7.600 ± 0.4879 mmol/L in HFD + FA group, *p* < 0.05) (Figure 6C). Furthermore, metabolic cage studies reveal that FA-treated mice have elevated energy expenditure elevated energy expenditure by 11.5% during dark phases (Figure 6D). Taken together, these results indicated that FA supplementation attenuates hyperglycemia and increases energy expenditure.

## Discussion

The synthesis of BAs is the most important mechanism for degrading and eliminating cholesterol. BAs are predominantly produced in the liver, *via* two biosynthetic pathways: The classical (or neutral) pathway and the alternative (or acidic) pathway (15). CYP7A1 as a key enzyme catalyzes the first and rate-limiting step in the classical BAs biosynthesis pathway (16). It was reported that *CYP7A1* deficiency in human results in a hypercholesterolemia and premature gallstone disease (23), and that mice overexpressing *Cyp7a1* have a decreased levels of plasma cholesterol (14, 24). Further analysis showed that the cholesterol-lowering effect has been ascribed to a decrease in the rate of LDL entry into the plasma space (25). In this study, we demonstrated that FA supplementation significantly increased the expression of *Cyp7a1* in liver (Figure 3). Although *Cyp7b1* mRNA level was also increased in HFD + FA group, its overexpression or deletion was reported to have no effect on BAs synthesis rates and plasma cholesterol levels (26, 27). Therefore, we hypothesized that FA attenuates HFD-induced hypercholesterolemia by activating *Cyp7a1*. Subsequently, by utilizing metabolomics, we found that two primary BAs in liver were significantly increased in HFD + FA group. These results suggest that FA supplementation increases the conversion of cholesterol to BAs by activating *Cyp7a1*.

It was notable that FA supplementation also significantly elevated fecal total BAs (Figure 1E). This observation resembled a previous finding that FA supplementation increases the content of fecal cholate and acidic sterol in *ApoE*^–/–^ mice (12) and male rats (13), respectively. Therefore, it is likely that FA activates *Cyp7a1* in mice, which may lead to the increases of BAs excretion subsequently. However, the opposite mechanism may also work: FA treatment could cause the increases of BAs excretion, which could subsequently lower their concentration forcing the upregulation of the hepatic *de novo* synthesis of BAs from endogenous cholesterol by activating *Cyp7a1* (28). This mechanism appears to apply in the effect of cholestyramine (29). One of the differences between the two mechanisms is the changes of BAs reabsorption. In our results of serum targeted metabolomics and qRT-PCR of ileum tissue, FA supplementation increases the size of serum BAs pools and the mRNA level of BAs transporter including OSTα and ASBT (Figure 4C), indicating that FA supplementation increases intestinal BAs reabsorption. These results arguing that FA activates *Cyp7a1*, which lead to the subsequent increase of BAs excretion. In other words, FA reduces serum cholesterol by activating CYP7A1 rather than increasing the excretion of BAs in feces.

FXR is responsible for maintaining BAs homeostasis in gastrointestinal tract (30). The previous research revealed a gut-liver-microbiome axis in which intestinal microbiota affects BAs biosynthesis by regulating the expression of *Cyp7a1* in the liver via a FXR-dependent mechanism (31). For example, the decreased abundance of BSH-enriched gut bacteria directly reduced BSH activity to elevate ileal conjugated BAs, which then inhibited FXR while decreased the expression of FGF15 in the ileum (32–34). In our study, FA supplementation did not alter the total abundance of BSH-enriched bacteria in fecal microbiota (Figures 5C, D) and the mRNA levels of hepatic SHP and ileal FGF15 (Figure 5E). These results suggest that FA activates *Cyp7a1* through non-FXR signaling. Interestingly, further microbiome analysis revealed an FA-induced moderate microbiota shift with significant changes of several specific bacteria genera, among which the relative abundance of one taxon of *Eisenbergiella* increased from 0.03 to 8.83% after mice were treated by FA (Supplementary Figure 4). it was reported that the intake of polyphenols anthocyanins and genistein also increased the abundance of *Eisenbergiella* (35, 36), suggesting its presence may be associated with benefit of polyphenols.

The gut bacterial genus *Eisenbergiella* belongs to the family *Lachnospiraceae* of the phylum *Firmicutes* and is strictly anaerobic. *Eisenbergiella* is commonly emerging in mice and humans subjected to carbohydrate-low diet interventions and is highly correlating with serum concentrations of 3-hydroxybutyrate, a ketone body produced through fatty acid β-oxidation (37). In this study, the enrichment of *Eisenbergiella* in gut microbiota indicated increased β-oxidation of fatty acid, which may be associated with the anti-obesity potential of FA. Of note, some species of genus *Eisenbergiella* were found to be bile-tolerant (38, 39). Therefore, it is possible that the enrichment of *Eisenbergiella* in the intestinal tract of the FA-treated obese mice was due to the combined effects of HFD and accumulated BAs.

Furthermore, the *Cyp7a1* transgenic mice were resistant to HFD-induced insulin resistance and obesity. Likewise, the FA-treated mice had a decreased fasting glucose level and an increased whole body energy expenditure (Figure 6), consistent with previous reports (40–42). Mechanistically, increased BA biosynthesis leads to elevated levels of circulating BAs (43) that then activates BA receptors in extrahepatic tissues to exert beneficial effects. Taking TGR5, one of BA receptors as an example. In pancreatic β cells, TGR5 activation will promote the release of insulin to improve the glucose metabolism (44). In skeletal muscle and brown adipose tissue, TGR5 activation will increase energy consumption by promoting the conversion of inactive thyroxine (T_4_) into active thyroid hormone (T_3_). Then the increase of lipids used as the energy substrate reduces lipids transport from extrahepatic tissues to hepatic tissues. Therefore, these beneficial effect may be attributed to the role of BAs as signaling molecules to regulate metabolism (36, 37).

However, we did not observe the upregulation of *Cyp7a1* or *Cyp7b1* in cultured mouse primary hepatocytes or human liver HepG2 cells following FA treatment (data not shown), suggesting that cellular responses to FA may vary greatly between the *in vivo* and *in vitro* systems, hence an extracellular signaling pathway may be required for the FA effect.

**FIGURE 7:**
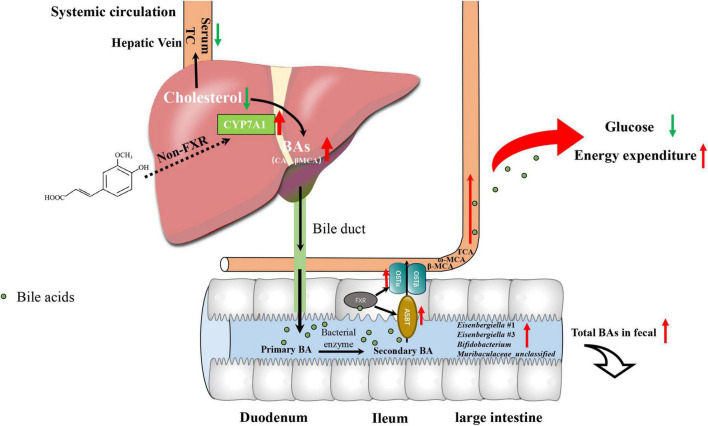
Proposed mechanism for the cholesterol-lowering effects of FA supplementation.

Taken together, we performed multi-omics approaches on microbiome, metabolome, gene expression and biochemical analysis to study the mechanism by which FA ameliorated hypercholesterolemia in HFD-fed mice and linked it to glucose and energy metabolism. The mechanism involves the following (Figure 7): (1) FA increased the expression of CYP7A1, a rate-limiting enzyme in BAs biosynthesis in the liver firstly via non-FXR signaling, which results in a decrease in serum TC level; (2) the level of BAs secreted to intestinal tract increased, resulting in the enrichment of the BA-tolerant gut bacteria *Eisenbergiella* in the intestinal tract and increased excretion of BAs in fecal samples; (3) increased serum BAs level activates BAs receptor of extrahepatic tissue, which lowers fasting glucose level and increased energy expenditure. These findings highlighted that FA could be considered as a powerful diet supplementation with potential hypocholesterolemic activity, which also supported the health benefits of a whole grain diet in hypercholesterolemia therapy.

## Data availability statement

The original contributions presented in this study are included in the article/Supplementary material, further inquiries can be directed to the corresponding author/s.

## Ethics statement

The animal study was reviewed and approved by the Institutional Animal Care and Use Committee of the Northwest A&F University.

## Author contributions

MW, LG, JB, and LH designed the project. ML performed most of the experiments. ZL analyzed the data and wrote the manuscript. FL and EE-O contributed to comments and revision in the manuscript. JY, YZ, and JL contributed to the animal study. All authors involved in editing the manuscript.

## Footnotes

1
https://www.kegg.jp/
