# Supplementary material for: Ferulic acid attenuates high-fat diet-induced hypercholesterolemia by activating classic bile acid synthesis pathway
Source: Front Nutr. 2022 Sep 21;9:976638. doi: 10.3389/fnut.2022.976638 (PMC9536491; doi:10.3389/fnut.2022.976638)
Supplement: Supplementary file 1 [file Data_Sheet_1.PDF]

# Figure S1

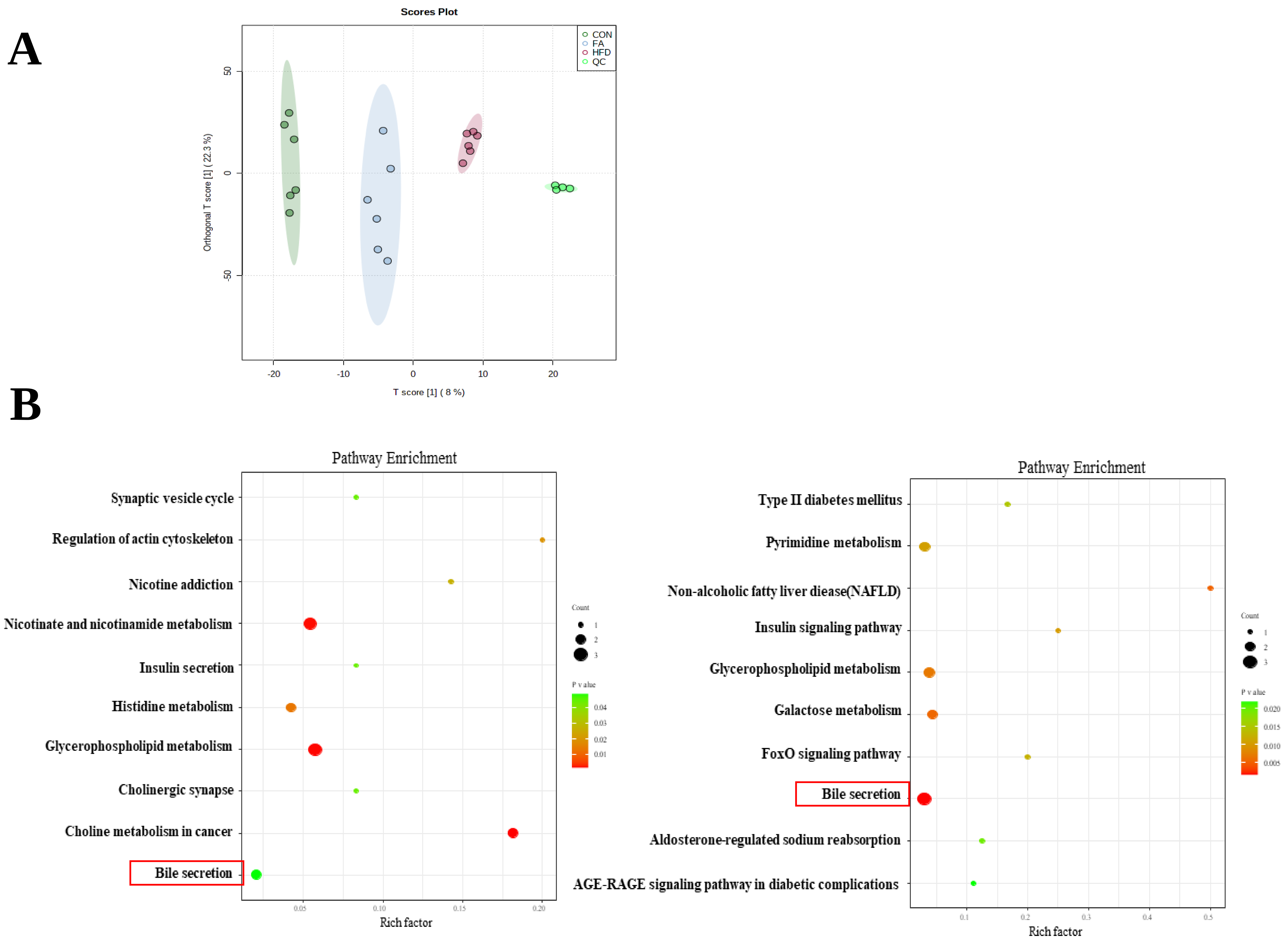


**Supplementary Figure 1. Effects of FA supplement on hepatic metabolite profile in HFD-fed mice.**

A) OPLS-DA score plots of the hepatic metabolomic profiles from the CON, HFD and HFD+FA mice. QC, quality control sample. B) KEGG pathway analysis based on significantly differential metabolites between groups: HFD versus CON (left) and HFD+FA versus HFD (right). CON: control diet group, HFD: high-fat diet group, HFD+FA: high-fat diet with 100 mg kg$^{-1}$ bw/day FA group.

# Figure S2

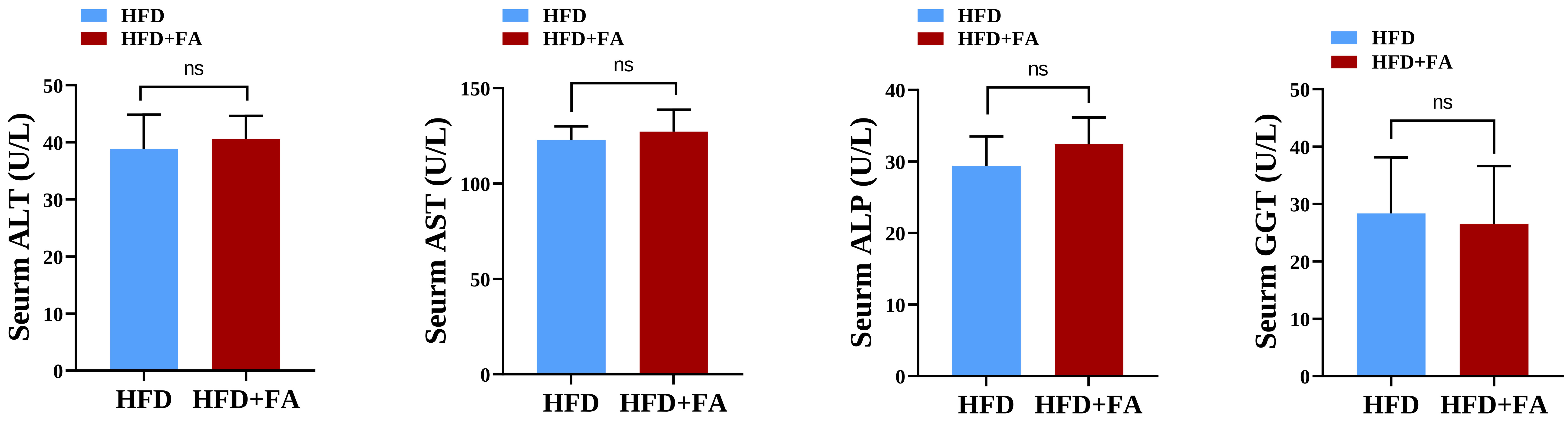


**Supplementary Figure 2. The serum ALT，AST，ALP and GGT levels of HFD and HFD+FA mice (n = 6).**

Data are presented as means ± SEM. ns, no significance. HFD: high-fat diet group, HFD+FA: high-fat diet with 100 mg/kg bw/day FA group.

# Figure S3

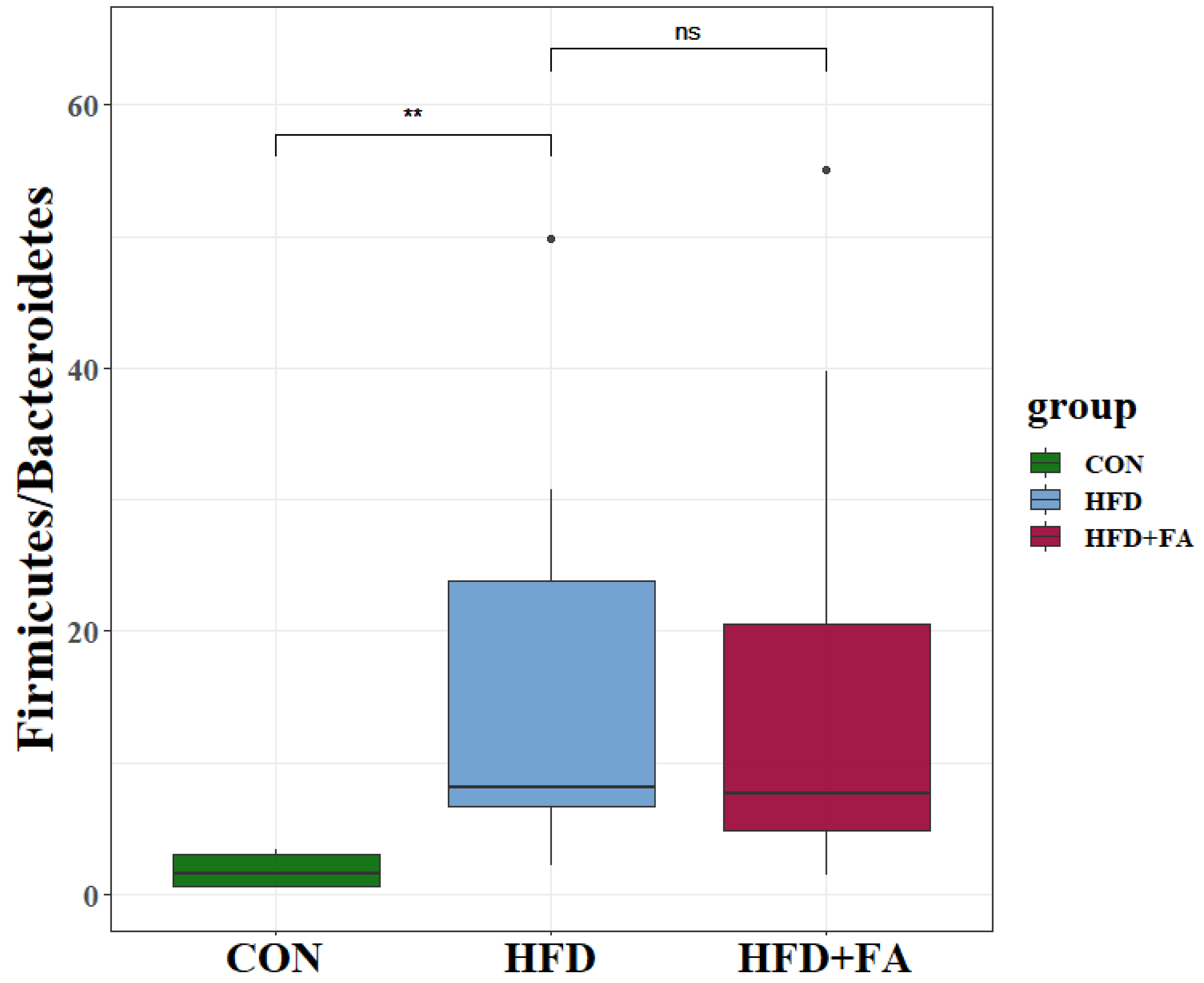


**Supplementary Figure 3. The ratio of Firmicutes/Bacteroidetes in HFD-fed mice (n = 8).**

Data are presented as means ± SEM.** $p < 0.01$, ns, no significance versus HFD group. CON: control diet group, HFD: high-fat diet group, HFD+FA: high-fat diet with 100 mg/kg bw/day FA group.

# Figure S4

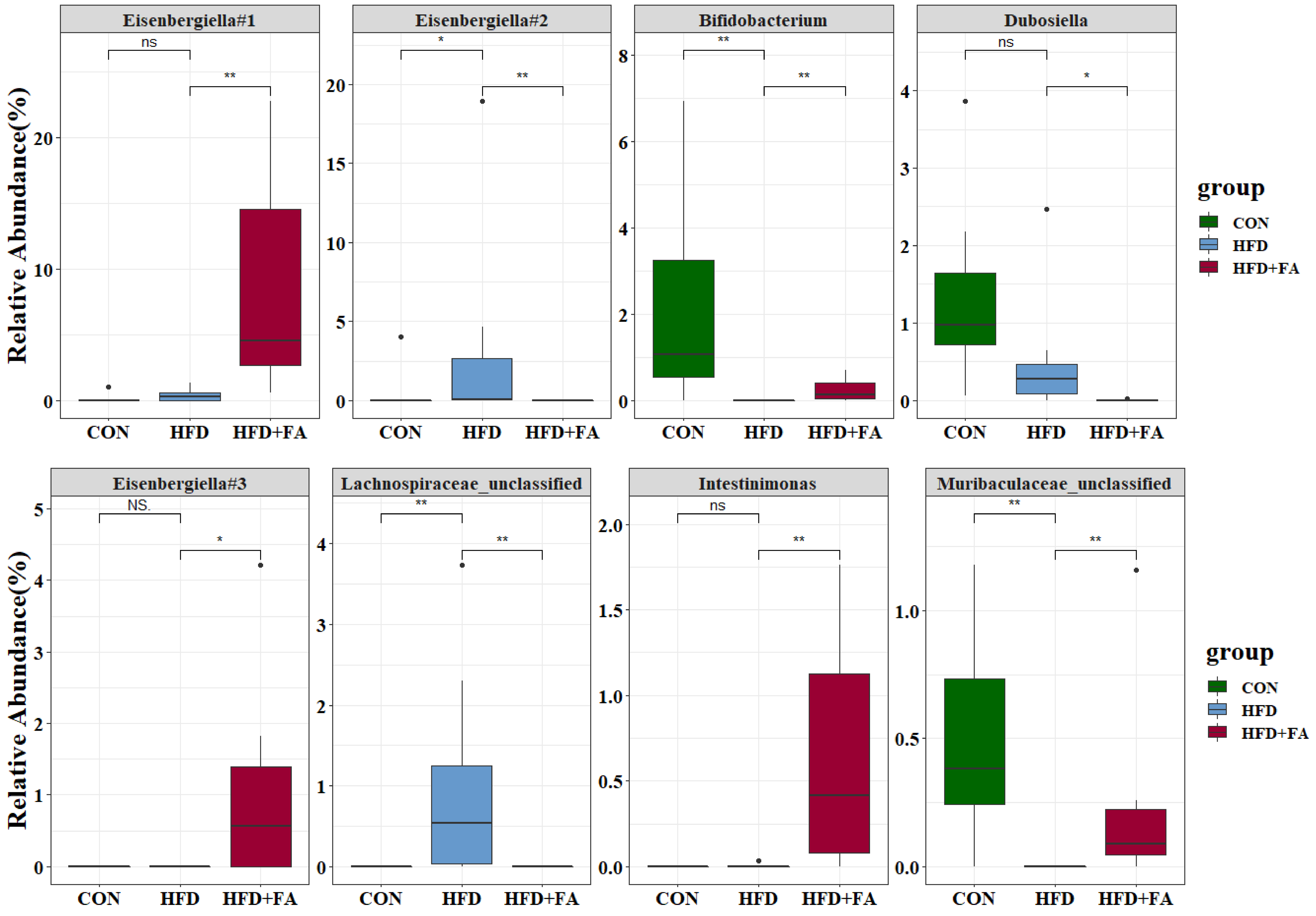


**Supplementary Figure 4. The abundance of 8 taxa at OTU level in three group mice (n = 8).**

Data are presented as means ± SEM. * $p < 0.05$, ** $p < 0.01$. CON: control diet group, HFD: high-fat diet group, HFD+FA: high-fat diet with 100 mg/kg bw/day FA group.
